# Thermal Analysis of Flip-Chip Bonding Designs for GaN Power HEMTs with an On-Chip Heat-Spreading Layer

**DOI:** 10.3390/mi14030519

**Published:** 2023-02-23

**Authors:** Kuo-Bin Hong, Chun-Yen Peng, Wei-Cheng Lin, Kuan-Lun Chen, Shih-Chen Chen, Hao-Chung Kuo, Edward Yi Chang, Chun-Hsiung Lin

**Affiliations:** 1 Semiconductor Research Center, Hon Hai Research Institute, Taipei 114699, Taiwan; 2 International College of Semiconductor Technology, National Yang Ming Chiao Tung University, Hsinchu 30010, Taiwan; 3 Department of Materials Science and Engineering, National Yang Ming Chiao Tung University, Hsinchu 30010, Taiwan

**Keywords:** bonding, flip-chip device, high-electron mobility transistor, thermal analysis

## Abstract

In this work, we demonstrated the thermal analysis of different flip-chip bonding designs for high power GaN HEMT developed for power electronics applications, such as power converters or photonic driver applications, with large gate periphery and chip size, as well as an Au metal heat-spreading layer deposited on top of a planarized dielectric/passivation layer above the active region. The Au bump patterns can be designed with high flexibility to provide more efficient heat dissipation from the large GaN HEMT chips to an AlN package substrate heat sink with no constraint in the alignment between the HEMT cells and the thermal conduction bumps. Steady-state thermal simulations were conducted to study the channel temperatures of GaN HEMTs with various Au bump patterns at different levels of current and voltage loadings, and the results were compared with the conventional face-up GaN die bonding on an AlN package substrate. The simulations were started from a single finger isolated HEMT cell and then extended to multiple fingers HEMT cells (total gate width > 40 mm) to investigate the “thermal cross-talk” effect from neighboring devices. Thermal analysis of the GaN HEMT under pulse operation was also performed to better reflect the actual conditions in power conversion or pulsed laser driver applications. Our analysis provides a combinational assessment of power GaN HEMT dies under a working condition (e.g., 1MHz, 25% duty cycle) with different flip chip packaging schemes. The analysis indicated that the channel temperature rise (∆T) of a HEMT cell in operation can be reduced by 44~46% by changing from face-up die bonding to a flip-chip bonding scheme with an optimized bump pattern design.

## 1. Introduction

GaN-based HEMTs demonstrate superior performance for applications in power electronics because of their outstanding material properties of a high breakdown electric field (1–3 MV/s), high electron saturated velocity (2.2 × 10^7^ cm/s), and high sheet electron density (above 1.0 × 10^13^ cm^−2^) [1]. A radio frequency (RF) GaN HEMT power amplifier with output power density more than 40 W/mm or a GaN HEMT power converter with breakdown voltage higher than 1200 V have been demonstrated [1,2]. More recently, GaN HEMTs have shown their capabilities in the emerging applications of high-power pulsed laser driver for the LiDAR applications [3].

For the packaging and integration of the GaN HEMT device on a package substrate, face-up die bonding with a wire connection and flip-chip bonding processes are frequently used techniques. Flip-chip technologies offer the major advantage of lower parasitic inductances over the face-up die bonding method by avoiding the wire connections [4]. Moreover, multiple active and passive devices can be integrated on a single substrate with all components being flip-chip bonded and communicating through the preformed thin/thick film metal conductors on the substrate to increase the compactness [5]. Connecting multiple active devices in parallel to raise the output current level is also a more effective way to reduce parasitic effects than a single large device [6]. Although flip-chip technology provides benefits to enhance the performance and to increase the density of integration, the high power GaN HMETs may suffer from self-heating as the power is increased and confined in a small area. Therefore, good thermal management through the packaging designs become a critical issue.

For the thermal management of the GaN HEMT packages for power applications including RF power amplifiers or power converters, highly thermal conductive package substrates, such as AlN or metal-coated ceramics, are usually employed as a heat sink [7]. For conventional face-up attached GaN device dies with the backside mounted on a heat sink, the heat dissipation is greatly affected by the wafer-type (e.g., Si, sapphire, etc.), the package substrate and the die attaching materials (i.e., epoxy, eutectic solder, etc.). By contrast, flip-chip packaging offers the capability to locate the metal bump to directly connect the face-down active region of the device with the heat sink and leads to much better thermal management. A variety of the flip-chip bonding schemes for a GaN HEMT RF power amplifier were reported, and the commonly recognized effective designs usually involved directly connecting the individual drain and source fingers with the AlN heat sink. Different bumps or underfill materials were also studied [8,9]. However, such a bonding scheme had smaller bump dimension and required high resolution in alignment.

To date, most of the designs and thermal analyses for the flip-chip package of GaN HEMT that are reported are based on RF power amplifiers (i.e., RF GaN HEMT), the device layout, dimension and operation modes of GaN HEMT for power management (i.e., power GaN HEMT) or photonic device drivers are quite different. First of all, to sustain the much higher operating drain voltages up to several hundred or kilo-volt ranges, the source-drain metal space (L_ds_) is much wider. The L_ds_ of a typical RF GaN HEMT is less than 5 µm, while L_ds_ for a power GaN HEMT is usually near 20 µm. That means the metal density of RF GaN HEMT is higher. Secondly, the total gate periphery (W_g_) of power GaN HEMT is much longer than RF GaN HEMT in order to handle to up to decades of amperes of current. The total W_g_ of an RF GaN HEMT is typically a few millimeters (mm), with each gate finger width limited within a few hundred micrometers (µm) for multiple gate devices [10] in order to handle high frequency signals. The total gate width of power HEMT is in the range of several tens up to hundreds of millimeters with a single gate finger width larger than 1mm. The larger gate periphery of power GaN HEMT also leads to a much larger die size. Thirdly, the RF GaN HEMT is operated under continuous DC bias, while power GaN HEMT is operated without DC bias with pulsed drain current conduction at a frequency of kHz to MHz ranges.

Previously, conventional face-up double gate fingers GaN HEMTs capped by a diamond heat spreader and a SiN passivation layer for RF application have been accomplished and discussed [11]. Thermal experiments show that thermal resistance of GaN HEMT with a diamond heat spreader layer is lower than traditional GaN HEMT with a SiN passivation layer by 21.4%. Furthermore, a diamond heat spreader has been applied on the Si hybrid micro-cooler for improving the hotspot’s cooling capability for RF GaN HEMTs [12]. The experiment and simulation results demonstrated that the estimated maximum temperature of the face-up GaN HEMTs can be reduced by 40.4% with a diamond heat spreader and 27.3% for that with a copper heat spreader, respectively.

In this paper, we focused our analysis on GaN HEMT to be used in power converter applications (i.e power GaN HEMT). We propose different flip-chip bump approaches to achieve good thermal performance for power GaN HEMT devices. Different from the direct connection of source-drain finger metal pads with an AlN heat sink using metal bumps for RF GaN HEMT, a metal heat-spreading layer was fabricated on top of the planarized dielectric layer above the active region of the HEMTs. Then, different Au heat-conducting patterns were used to connect the metal heat-spreading layer and the AlN packaging substrate heat sink. The design of the bump pattern was decoupled from the GaN HEMT layout to provide flexibility for designing bumping structures with high thermal efficiency and mechanical robustness. We compared thermal simulations among face-up die bonding and the proposed flip-chip bonding schemes using the finite element software COMSOL. Thermal simulations were conducted step-by-step from a single gate finger HEMT cell, and then extended to multiple fingers HEMTs with various finger amounts to study the “thermal cross-talk” effects [13]. The channel temperatures of the power HEMTs of various bonding schemes were first accessed based on steady state DC conditions in order to quickly collect the effects of various voltage, current and transistor peripheries. Then, the more time-consuming dynamic analysis based on the pulsed current that is close to the actual operating condition of power GaN HEMT was conducted at selected conditions to provide a comprehensive assessment of a power GaN HEMT dies under a working condition (e.g., 1 MHz, 25% duty cycle) with different packaging schemes [14]. We have also shown that by using the metal heat-spreading layer, as well as optimized bump design, the improved thermal dissipation allows up to 44~46% reduction in channel temperature rises (∆T) for multiple fingers GaN HEMTs compared with the face-up bonding scheme, and can be achieved for both steady state and pulse operations.

## 2. Device Structures for Thermal Analysis

**Figure 1:**
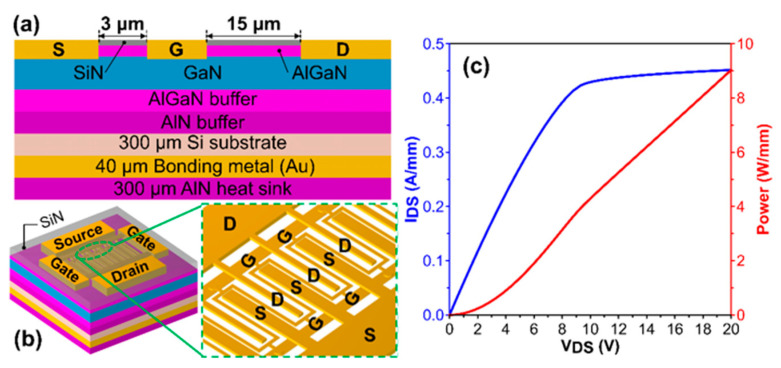
(**a**) Cross-sectional plot of a single GaN HEMT device. (**b**) The three-dimensional schematic of a multiple gate HEMT device. (**c**) The drain current–voltage curve and corresponding power density of a single GaN HEMT.

The AlGaN/GaN HEMT grown on a Si substrate was employed for the study. The heterostructure consists of AlGaN (25 nm), an undoped GaN layer (1.3 µm) and a GaN/AlGaN/AlN buffer layer (4 µm). Figure 1a depicts the cross-sectional structure of the GaN HEMT device. The HEMT device with gate offset had a gate length of 2.5 µm and a source-to-drain space of 20.5 µm. The ohmic contact to the gate distance were 3 µm and 15 µm for the source and drain, respectively. The layout of the multiple fingers HEMT for simulation is depicted in Figure 1b. For the simplicity of analysis, the gates were separated equally with a pitch size of 100 µm, and the sizes of the source and drain contact pads were adjusted to achieve the gate offset with a larger gate-to-drain space than gate-to-source space. The drain current–voltage (I–V) performances for the thermal simulation were obtained from a 20-fingers HEMT with a total gate width of 20 mm (i.e., each finger is 1 mm) with a constant gate voltage (e.g., 0 V), as shown in Figure 1c. The thickness of the Si substrates of the GaN chips were 300 µm. The power density of the heat generated is also labeled in Figure 1c. The channel temperature of the face-up bonded (FU-0) GaN HEMT device on an AlN heat sink using a Au bonding layer is simulated as the controlled reference to compare with our new flip-chip bonding design. 

**Figure 2:**
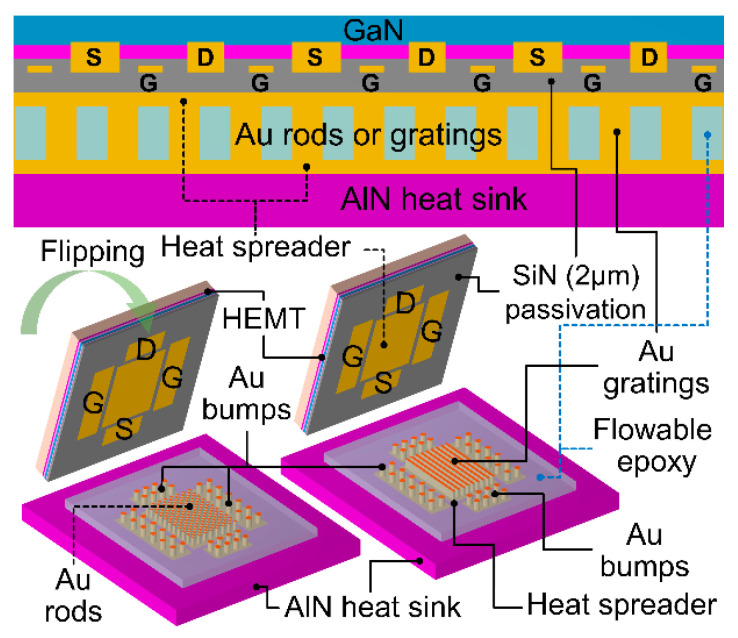
Cross-sectional view and three-dimensional schematics of the proposed flip-chip bonding designs for high power GaN HEMTs.

For the flip-chip bonded devices in this study, the schematic drawings are depicted in Figure 2. The scheme of flip-chip bonding with a heat spreader above the active region is different from most previously reported structures, which have their bumps for heat conduction directly connected between the ohmic contact pads and the AlN heat sink [8]. We inserted a Au heat spreading metal layer on top of the chip, which was covered by a planarized dielectric/passivation layer, for the Au bumps (i.e., Au rods/gratings) to connect between the chips and the AlN package substrate for heat conduction. A planarized silicon nitride (SiN) layer of 2 µm covered the HEMT as an interlevel dielectric layer and a passivation layer. Then, a heat-spreading metal layer (i.e., Au or Cu) was deposited on top of the SiN layer. The thick metal bump for electric connection or heat dissipation were formed on the 300 µm-thick AlN package substrate heat sink using the standard thick film fabrication processes, which are provided by many commercial suppliers. Then, the GaN device die and the AlN package substrate were bonded face-to-face using metal solders, high temperature hot press or ultrasonic methods. Two major types of metal bump patterns were employed. The first type (FC-1) consisted of circular-shaped Au rods with a diameter of 50 µm with a space between the pillars of 100 µm. The second type (FC-2) consisted of long stripes along the FET finger directions to form a grating structure with a width of 50 µm and pitch of 100 µm. The height of the metal bump was 36 µm. Two heat-spreading layers with a thickness of 2 µm were deposited on the chip and the package substrates, which indicates the total height for heat conducting between the chip and the package substrates was 40 µm.

## 3. Results and Discussion

The analysis of the thermal behavior of the different integration structures employed a three-dimensional (3D) finite element analysis model. The heat generated in the channel region based on the I–V behavior (as shown in Figure 1c) of the GaN HEMT is taken as the heat source. The heat is assumed to be generated along the channel region and the location of heat generation can be accurately predicted by the use of the two heat source model [15]. The material properties, including density (ρ), thermal conductivity (κ) and heat capacity (C_p_) for the thermal analysis are listed in Table 1. Typically, the thermal properties of materials are temperature dependent, therefore, the material nonlinear effects [16] were also included in our analysis and the parameters for the temperature of 300 K used in the simulation are taken from Refs. [17,18,19,20,21]. In addition, recently, the comparison of linear and nonlinear thermal conductivity models for AlGaN/GaN HEMTs has demonstrated that the calculated data of the nonlinear model are in good agreement with the measured data [22].

The thermal interaction between the individual gate fingers for the parallel-connected multiple gate HEMTs, which is known as the “thermal cross-talk” effect, were also included in our analysis. Such effect is very significant and cannot be neglected for the power GaN HEMT with large gate periphery. As mentioned previously, the W_g_ of power GaN HEMT can be in the range of tens to hundreds of millimeters with tens of HEMT cells connected in parallel (i.e., multi-finger HEMTs). The accumulation of heat transmitted from neighboring parallel HEMT cells of a specific HEMT cell could raise the channel temperature significantly. However, the temperature rise owing to the thermal cross-talk effect would become saturation as the parallel HEMT cell amount (i.e., number of fingers) reached larger numbers due to thermal equilibrium between heat generation and heat dissipation by the whole structure. The saturation of the channel temperature rising with respect to the amounts of parallel HEMT cells may not be observed for smaller RF GaN HEMT dies. 

Moreover, the thermal analysis based on the steady state can be more straightforward; nevertheless, transient analysis for devices under pulse operation is necessary for reflecting the thermal behavior under many actual use conditions. For GaN HEMT used in power electronics, there is usually no steady state DC bias. Typically, the GaN HEMT is operated in pulsed drain current conduction mode, in which the peak power could be much higher than the average power, while the duty cycle may be low and adjustable, at a frequency of kHz to MHz ranges. The channel temperature rises and falls following the drain current pulses. However, the temperature would not fall back to the original point right away for each cycle, and the time-dependent transient behavior in heating and cooling during an operating cycle need to be well analyzed. The temperature rise in pulse operation is the accumulation of the residual heating from previous cycles. The steady-state thermal simulation would result in an overestimated channel temperature rise. Nevertheless, such steady-state analysis provides an upper bounding situation. Additionally, it can also be used to estimate the pulse current condition after being adjusted to a derated steady state average power. Our thermal analysis was started with steady-state analysis for a single HEMT cell, then we extended the simulation to verify the thermal cross-talk effect of multiple HEMT cells. Thermal simulations of GaN HEMT under pulse bias and current, which mimics the real situation of operation of a converter or pulse laser driving circuit, were also conducted to analyze the channel temperatures of different packaging scheme.

**Figure 3:**
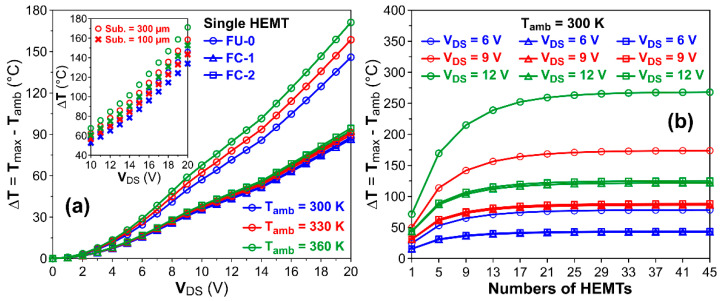
Calculated channel temperature rises for (**a**) single gate and (**b**) multiple gate GaN HEMTs. The channel temperature rises of FU-0, FC-1 and FC-2 are indicated by the circle, triangle and square, respectively. The blue, red and green lines plotted in Figure (**a**) indicate that the ambient temperatures are set to 300 K, 330 K and 360 K, respectively.

Figure 3a shows the comparison of the channel temperature rise (ΔT = T_max_ − T_amb_) of a single gate HEMT cell using different bonding schemes (FU-0, FC-1 and FC-2) under different DC bias conditions, as well as different ambient temperatures, T_amb_ (i.e., 300 K~360 K), where T_max_ indicates the maximum channel temperature. It can be seen that the FU-0 device showed the higher ΔT, with values ranging from 145.9 °C to 171.1 °C for ambient temperature, and ranging from 300 K to 360 K with V_DS_ up to 20 V (i.e., drain current saturated at 0.45 A/mm). The sensitivity to ambient temperature could be to the result of a relatively longer heat transfer distance of the FU-0 scheme and lower thermal conductivity resulting from the effect of nonlinear thermal conductivity. For the FC-1 bonding scheme, the temperature rise can be decreased down to 86.4 °C to 92.6 °C with minor dependence on ambient temperature, while the FC-2 showed a similar temperature rise near 87.8 °C to 94.4 °C. It is very clear that the shorter distance from the heat generating channel to the AlN heat sink of the flip-chip bonding schemes (i.e., bump height) leads to the cooler channel temperature and less sensitivity to ambient temperature than the FU-0 scheme. The slightly difference (<2 °C) in channel temperatures between FC-1 and FC-2 are more likely owing to the different spatial overlapping of the Au bump patterns and GaN HEMT fingers. The bump pattern with Au gratings in the simulation are not deliberately aligned to the multiple fingers of the GaN HEMT devices in our study. If the arrangement of long grating patterns were purposely designed to align with the FET source/drain fingers, FC-2 could probably provide more efficient heat transfer paths than the rod-shaped bumps of FC-1. The inset of Figure 3a also compares the channel temperatures for the FU-0 with the substrate thickness of 100 µm and 300 µm, and the difference in the channel temperature is around 11.9~18.4 °C for V_DS_ = 20 V under ambient temperatures between 300 K to 360 K. The results suggest that the flip-chip bonding for the GaN HEMT chip with a heat-spreading layer showed superior heat dissipating capability than the FU-0 structures. Figure 3b demonstrates the channel temperature rises (ΔT) of multiple fingers HEMTs under different drain bias (V_DS_) of 6 V, 9 V and 12 V for T_amb_ = 300 K, different from the drain current (i.e., as shown in Figure 1c) to illustrate the “thermal cross-talk” effects. The channel temperature rises are plotted against different amounts of gate fingers. It can be seen that the both FC-1 and FC-2 flip-chip bonding schemes showed lower ΔT than the FU-0 case. The ΔT increased with the parallel connected gate amounts and gradually became saturated above 21 and 13 HEMT cells stacked for the FU-0 and flip-chip bonding schemes when V_DS_ = 12 V. The saturation shifted to more gate amounts as the drain voltage increased, indicating more lateral heat conduction with a hotter channel. The discrete RF device structures with various gate fingers (≤10 fingers) amounts and different gate pitches between 25 µm to 50 µm were reported previously [23,24]. The typical unit gate width is below 500 µm per gate. In this study, we extend the analysis to GaN HEMTs for power conversion or photonic device driver applications, and the HEMT dimensions are in the range of tens of millimeters. We simulated the device with a unit gate finger width of 1 mm and a gate pitch of 100 um, with amounts of the gate up to 45. It can be seen in our study that the channel temperature rising can increase around 3.2 to 3.7 times for the FU-0 structure when the drain bias raises from 6 V to 12 V by comparing the single gate ΔT value and the saturated multiple gate ΔT values. For example, the channel temperature can increase from 71.6 °C to 268 °C for FU-0 structures biased at 12 V as the gate amounts increase from 1 gate to 45 gates. On the other hand, the channel temperature rise of the flip-chip bonded structures can be limited below 3 times between ΔT of a single gate and saturated ΔT of multiple gate devices. For example, the channel temperature increased from approximately 43.4 °C to 121.9 °C for the FC-1 structure biased at 12 V as the gate amount increased from 1 gate to 45 gates. By utilizing of the FC-1 and FC-2 structures, the saturated ΔT for multiple fingers HEMTs can be dramatically decreased to 45.5% and 46.7% of ΔT of a conventional face-up bonded (FU-0) structure. 

For RF GaN HEMT, there are several thermal analysis reports published by manufacturers [25,26]. To further compare the thermal performances between our analysis on power GaN HEMT and the reported RF GaN HEMTs, the channel temperature can be compared for face-up mounted dies so as to exclude the effects of flip-chip bump designs. For example, the measured channel temperature rise (ΔT) of 157 °C was reported for a multiple gate finger 14.4 mm gate width (W_g_) HEMT with 4 W/mm power dissipation [25]. The channel temperature rise reported is close to our thermal simulation results of ΔT ~150 °C for 13 fingers HEMT (W_g_ = 13 mm, Figure 3b), though the substrate and die attaching solder are different. On the other hand, the RF GaN HEMT with a smaller W_g_ of 2 mm exhibiting a lower temperature rise of approximately 70~80 °C was reported [26], which is near our results for 2 fingers HEMT (W_g_ = 2 mm, Figure 3b). The lower temperature rise could be attributed to the weaker “thermal cross-talk” effect of the smaller HEMT size. Based on the comparison, we suggest that the thermal performances are similar between the reported RF GaN HEMT and our power GaN HEMT, regardless of the differences in the device layouts (i.e., L_ds_, L_g_ and gate pitch).

**Figure 4:**
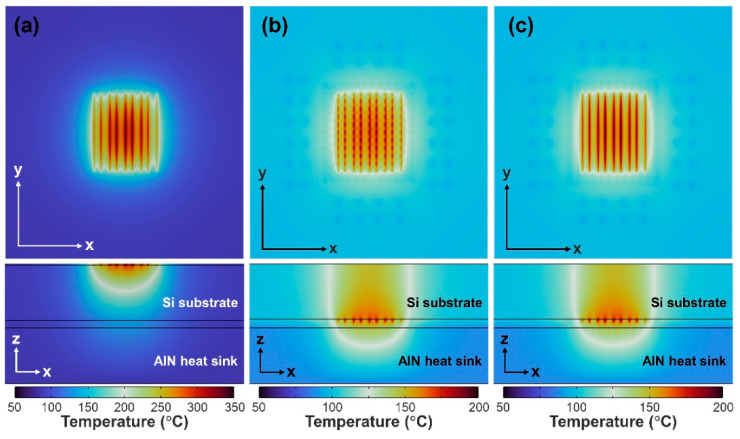
Temperature distributions of GaN HEMTs with nine gate fingers operated at V_DS_ = 12 V and T_amb_ = 360 K for (**a**) FU-0, (**b**) FC-1 and (**c**) FC-2 bonding schemes. The x–z cross-section is cut along the center of the AlGaN channel.

The temperature distributions in the x–y and x–z cross-section plots of GaN HEMTs with nine gate fingers operated at V_DS_ = 12 V and T_amb_ = 360 K for three different bonding schemes are depicted in Figure 4. For the FU-0 scheme, the simulated temperature distribution is shown in Figure 4a. The heat was generated at the channel, then radially spread from the center of the channel to the heat sink through the Si substrate and bonding metal. For the flip-chip bonding schemes, the heat would be spread to both upward and downward paths. The upward heat flow is dissipated through Si substrate to the air, while the downward heat flow is conducted through the Au metal heat-spreading layer and epoxy/Au bumps to the AlN package substrate heat sink. The relevant temperature profiles are demonstrated in Figure 4b,c for the FC-1 and FC-2 schemes, respectively. Furthermore, a material with high thermal diffusivity, α = κ/(ρ·C_p_) [27], means that the heat can be rapidly transferred from the hot region to the cold region. The estimated thermal diffusivities are also listed in Table 1. In spite of containing epoxy, which has worse thermal conductivity and diffusivity properties, the average thermal diffusivities (44.6 mm^2^/s and 58.4 mm^2^/s) of whole flip-chip package bodies including Au heat-spreading layers, epoxy and Au rods/gratings patterns for FC-1 and FC-2 are approximately in a similar magnitude with the thermal diffusivity value of GaN. The thinner package body of flip-chip bonding schemes with Au heat-spreading layers would be beneficial for the quicker relief of the thermal accumulation.

**Figure 5:**
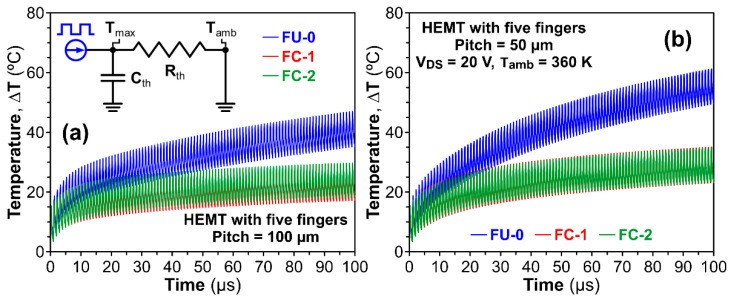
Transient temperature responses of three different bonding schemes. The GaN HEMTs with five gate fingers operated at a pulse-width modulation frequency of 1 MHz with a duty cycle of 25% for the gate pitch of (**a**) 100 µm and (**b**) 50 µm, respectively. The V_DS_ and T_amb_ are fixed at 20 V and 360 K. The inset of Figure (**a**) shows the effective thermal RC network model.

For power GaN HEMT, the applications in power conversion or driver for a photonic device (i.e., laser, light-emitting diode) are functioning in pulse mode. Thus, the transient heating effect of GaN HEMT under pulse operation was also analyzed. Figure 5 illustrates the transient temperature responses of GaN HEMTs with five gate fingers (i.e., W_g_ = 5 mm) for the three different bonding schemes. The power GaN HEMTs were simulated under more stringent conditions, e.g., V_DS_ = 20 V and T_amb_ = 360 K, to imitate the real working environment. The dynamic thermal simulation of a GaN HEMT device bonded on an AlN package substrate heat sink can be simplified as an equivalent RC circuit model, which consists of a series thermal resistance R_th_ and a shunt thermal capacitance C_th_, as shown in the inset of Figure 5a. The heating pulse was assumed to be a square wave with a pulse-width modulation frequency of 1 MHz (the corresponding time period is 1 µs) and a duty cycle of 25%. Figure 5a depicts the transient temperature rise of a five-fingers GaN HEMT device with a gate pitch of 100 µm being continuously operated for 100 pulses. Obviously, the calculated temperature ripples for the FU-0, FC-1 and FC-2 structures illustrate a clear difference. The ΔT are 41.1 °C, 23.1 °C and 24 °C for FU-0, FC-1 and FC-2, respectively. Moreover, as shown in Figure 5b, the ΔT for FU-0, FC-1 and FC-2 are increased from 41.1 °C to 55.4 °C, 23.1 °C to 29.1 °C and 24 °C to 29 °C, respectively, for the case of gate pitch = 50 µm. It is clearly demonstrated that the thermal cross-talk effect is still crucial for power GaN HEMT with multiple fingers operated at a higher modulation frequency. Through the steady-state and transient thermal analysis, it was shown that our proposed flip-chip bonding designs possess excellent capabilities to effectively dissipate the heat.

## 4. Conclusions

We have presented the steady state and transient thermal analysis of GaN HEMT with a Au metal heat-spreading layer flip-chip bonded on an AlN package substrate heat sink. The superior heat dissipating capabilities of the proposed flip-chip bonding schemes for power GaN HEMT were presented and compared with the conventional face-up bonding scheme. Analysis of the thermal cross-talk effect indicated that multiple gate HEMT could show 3.2~3.7 times higher channel temperature rising than single gate HEMT, depending on the various bonding schemes. The transient analysis for the pulse operation of the GaN HEMTs were also provided. The ΔT for FU-0, FC-1 and FC-2 increased 41.1 °C, 23.1 °C and 24 °C, respectively, for the case of gate pitch = 100 µm after continuous operation for 100 cycles with a frequency of 1 MHz and a duty cycle of 25 %. This work offers viable flip-chip bonding designs to provide better thermal management for power GaN HEMT.

## Figures and Tables

**Table 1:** Material constants used in the simulation for the temperature of 300 K are listed below.

Property	SiN	GaN	AlGaN	AlN	Si	Au	Epoxy
ρ (κγ/μ^3^)	3100	6150	6282	6810	2330	19,300	2080
κ (W/m·K)	18.5	130	17.2	180	148	310	1.22
C_p_ (J/Kg·K)	709	431	494.4	748	255	137	1010
α (μμ^2^/σ)	8.4	49	5.5	35.3	249.1	123.6	0.58

## Data Availability

Not applicable.
